# Strength gradient enhances fatigue resistance of steels

**DOI:** 10.1038/srep22156

**Published:** 2016-02-24

**Authors:** Zhiwei Ma, Jiabin Liu, Gang Wang, Hongtao Wang, Yujie Wei, Huajian Gao

**Affiliations:** 1 LNM, Institute of Mechanics, Chinese Academy of Sciences, Beijing 100190, P.R. China; 2 Faculty of Engineering, Zhejiang University, Hangzhou 310027, China; 3 Laboratory for Microstructures, Shanghai University, Shanghai 200444, P.R. China; 4 School of Engineering, Brown University, Providence, RI 02912, USA

## Abstract

Steels are heavily used in infrastructure and the transportation industry, and enhancing their fatigue resistance is a major challenge in materials engineering. In this study, by introducing a gradient microstructure into 304 austenitic steel, which is one of the most widely used types of stainless steel, we show that a strength gradient substantially enhances the fatigue life of the material. Pre-notched samples with negative strength gradients in front of the notch’s tip endure many more fatigue cycles than do samples with positive strength gradients during the crack initiation stage, and samples with either type of gradient perform better than do gradient-free samples with the same average yield strength. However, as a crack grows, samples with positive strength gradients exhibit better resistance to fatigue crack propagation than do samples with negative gradients or no gradient. This study demonstrates a simple and promising strategy for using gradient structures to enhance the fatigue resistance of materials and complements related studies of strength and ductility.

Property gradients have been used in materials to effectively enhance various mechanical properties, including wear, friction, corrosion resistance, thermal resistance, strength and ductility^1^,^2^,^3^. Such materials are ubiquitous in biological systems as a result of natural selection for performance optimization^3^,^4^,^5^,^6^,^7^. Materials with internal gradients in their Young’s moduli and/or strengths exhibit beneficial effects such as crack shielding, alleviation of stress concentrations, and reduced shear localization^1^,^2^,^3^. A sharp gradient in the grain size^8^,^9^,^10^,^11^ introduced by a processing technique called surface mechanical attrition treatment was found to increase the strength of polycrystalline copper without sacrificing its ductility. By applying a torsion-induced plastic deformation gradient to a cylindrical sample of twinning-induced plasticity (TWIP) steel in the radial direction, Wei *et al.*^12^ demonstrated that a linearly graded twin density can be introduced and used to enhance the tensile strength of a material without sacrificing its ductility. This pre-torsion treatment of materials can serve as a simple strategy for introducing linear gradients in properties on engineering scales and for fabricating samples to explore the fundamental properties of such materials.

The fatigue properties of metals are of enormous practical interest because over 90% of metallic engineering structures ultimately fail due to fatigue^13^,^14^. However, to date, there have been few studies of the fatigue behaviour of gradient materials. The residual stress at the surface layer may play an important role in the enhanced fatigue endurance of surface mechanical attrition treated samples with sharp surface gradients^15^,^16^,^17^,^18^. In this study, we consider linearly graded samples of 304 austenitic steel, which is one of the most widely used types of stainless steel, which exhibits both deformation twinning and martensitic transformation during plastic deformation^15^,^19^. The same pre-torsion treatment^12^ is used to prepare graded samples with linearly graded microstructures and initial strengths. Our attention is focused on how the fatigue performance of the material is enhanced by positive or negative strength gradients in front of a pre-existing crack.

## Results

**Figure 1:**
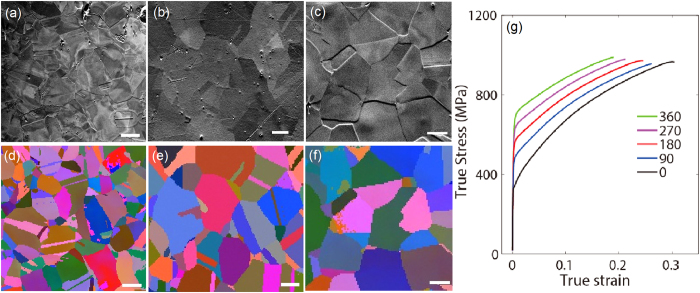
The torsion-induced gradient structure in 304 steel. (**a–f**) SEM images and corresponding EBSD pictures at different locations (scale bars 10 μm): (**a**,**d**) *r* = *R*; (**b**,**e)** *r* = *R*/2; (**c**,**f**) *r* = 0. (**g**) The tensile stress-strain behaviour of the pre-twisted and as-received samples.

Cylindrical bars of 304 stainless steel with a linearly graded microstructure comprising both deformation twins and martensitic transformations along the radial direction were fabricated by subjecting the as-received samples with no perceivable microstructure gradients to a previously developed pre-torsion treatment procedure^12^. Figure 1a–c shows scanning electron microscopy (SEM) photographs of selected locations from the centre to the surface of a sample subjected to 180 degrees of pre-torsion. The corresponding electron back-scattered diffraction (EBSD) images are shown in Fig. 1d–f. It was observed that pre-torsion enhanced the yield strength of 304 steel, increasing it from 330 MPa in the as-received state to 610 MPa following 180 degrees of pre-torsion and to 720 MPa following 360 degrees of pre-torsion. The increase in tensile strength came at the expense of a slightly reduced failure strain. The evolution of the microstructure and the strengthening effect of pre-torsion in 304 steel are consistent with previous findings on TWIP steel^8^,^12^. The twin volume fractions in the three areas shown in Fig. 1a–c were approximately 9%, 4% and 1%, respectively.

**Figure 2:**
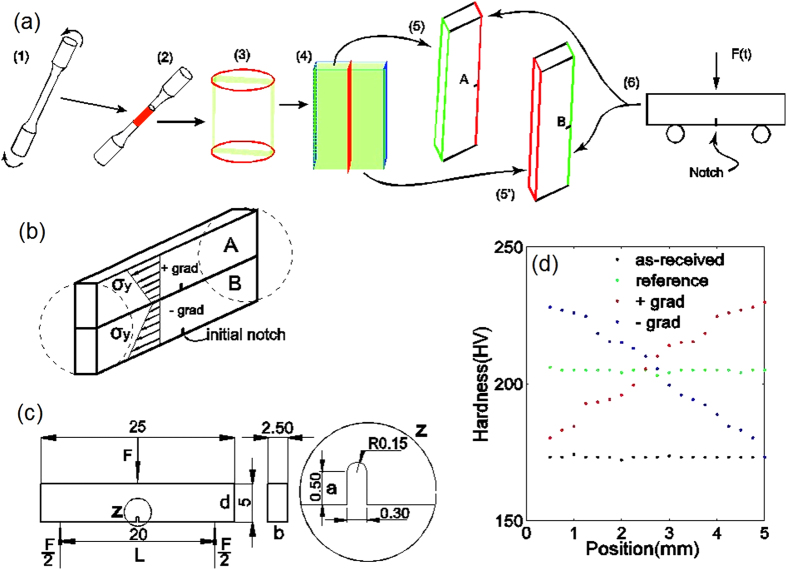
Graded sample preparation. (**a)** An illustration of the steps used to create the graded samples. (1) Applying pre-torsion to an as-received sample; (2) cutting a rod from the middle section of a pre-twisted sample; (3) sectioning the middle section of the rod; (4) splitting the plate into two parts from the middle; (5) making a positively graded sample by cutting a round-tipped crack in the side of the core with a lower yield strength; (6) making a negatively graded sample by generating a round-tipped crack on the surface side; and (7) performing an *in-situ* three-point bending test. (**b**) An illustration showing the positions of graded samples ‘A’ and ‘B’ in the pre-twisted bar and the dimensions of the fatigued samples used in the three-point bending tests (units: mm). (**c**) The hardness profile in the crack plane of the four types of sample: as-received gradient-free (as-received), pre-tensioned strengthened gradient-free (reference), positively graded (+grad), and negatively graded (-grad).

Then, the pre-twisted bars were used to create graded samples for fatigue tests. Readers are referred to the Methods section for details of the preparation of the graded samples. An illustration of the steps in that process is shown in Fig. 2a. When an initial crack was cut in a sample from the core side of a pre-twisted cylinder, as in sample ‘A’ in Fig. 2b, we obtained a sample with a positive gradient in which the initial yield strength increases in front of the crack tip. When the initial crack was located at the surface of the cylinder, as in sample ‘B’ in Fig. 2b, we obtained a sample with a negative gradient. In Fig. 2c, we provide the final dimensions of the fatigued samples used in three-point bending tests. The two types of graded sample had the same average yield strength. This average strength was greater than the yield strength of the as-received samples. To explore the effects of gradients, we also applied pre-tension to several as-received samples to make their initial yield strengths identical to the average yield strength of the graded samples. We labelled the samples with higher yield strengths than the as-received samples without strength gradients ‘reference’. Both the as-received samples and the reference samples were gradient-free. The crack plane hardness profiles of the four types of sample (negative, positive, as-received, and reference) are shown in Fig. 2d. Subsequently, we applied force-controlled cyclic loading (from 78.2 to 782 N) to the samples used in the three-point bending tests (see Methods and supplementary Fig. 1) during SEM observations. This load amplitude ensured that fatigue fracture occurred within a reasonable number of cycles and allowed us to complete one *in-situ* three-point bending test per day.

**Figure 3:**
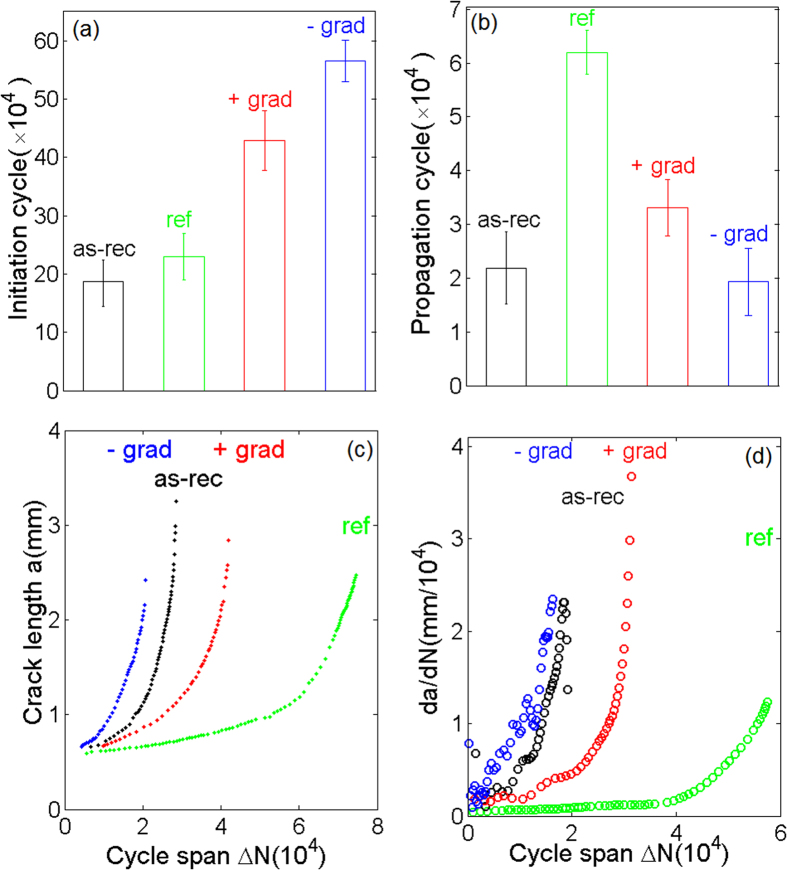
The fatigue properties of graded 304 steel under force-controlled bending. (**a,b**) The fatigue lives of the four types of sample: as-received gradient-free (as-rec), pre-tensioned gradient-free (ref), positively graded (+grad) and negatively graded (-grad): The number of cycles required for (**a**) crack initiation and (**b**) crack propagation (the error bars represent the standard deviations of three samples). (**c**) The crack length as a function of the number of loading cycles during the crack propagation stage in the experiments. (**d**) The derived crack propagation rate as a function of the number of loading cycles based on the experimental data in (**c**).

Because the round-tipped notch encouraged the initiation of a sharp fatigue crack, the overall process of fatigue fracture was divided into the following stages: crack initiation, steady-state crack growth, and rapid crack propagation until failure. The number of cycles leading to the formation of a sharp crack observable via SEM is defined as the life for crack initiation. Once it was initiated, the crack grew steadily in response to the cyclic load until final rupture of the sample, which happened quickly. All of the cycles after crack initiation were counted in the crack propagation life. Figure 3 depicts the fatigue performances of graded and gradient-free samples. A number of striking features are associated with the crack initiation and propagation stages in the four types of sample:

- In the crack initiation stage, the negatively graded sample experienced a total of *C*_*N*_ = 5.7 × 10^5^ loading cycles, while the positively graded sample experienced *C*_*P*_ = 4.3 × 10^5^ cycles. In comparison, the two gradient-free samples — the as-received sample and the reference sample — endured *C*_*1*_ = 1.9 × 10^5^ and *C*_*2*_ = 2.1 × 10^5^ cycles, respectively, in the crack initiation stage, as shown in Fig. 3a,b.
- In the crack propagation stage, the crack in the gradient-free reference sample propagated the slowest, and the crack in the positively graded sample was second. The crack in the negatively graded sample propagated the fastest (Fig. 3c,d).

The observation that the negatively graded sample exhibited a longer fatigue life during the crack initiation stage is consistent with the general observation of Fleck *et al.* that the fatigue limit is proportional to the yield strength of a gradient-free polycrystalline metal^20^. That the positively graded sample survived more fatigue cycles than the as-received gradient-free one is intriguing because the yield strength in the vicinity of the crack tip was similar for the two samples. After crack initiation, we saw that the gradient-free sample strengthened by pre-tension endured more cycles before fatigue fracture than the other three samples did (Fig. 3b), which indicates that a higher yield strength helps retard crack propagation.

To identify the factors governing the distinct fatigue properties of crack initiation and propagation in those four types of sample, we examined the fracture surfaces of the fatigued samples. As shown in supplementary Fig. 2, the fracture surface exhibited three characteristic regions commonly seen in stainless steel: the mirror, mist and hackle regions. These regions are labelled ‘c’, ‘d’, and ‘e’ in supplementary Fig. 2b, which depicts the negatively graded sample. Amplified SEM images of the three regions are shown in supplementary Fig. 2c–e. Transgranular cracking appears during the initiation stage and is visible in the side view of the crack (supplementary Fig. 2f). This result is consistent with general observations of the transition from transgranular failure combined with slow crack propagation to intergranular cracking combined with fast crack growth in 304 steel^21^. However, no apparent differences in the fractographies of the three types of sample (positively graded, negatively graded and gradient-free) were observed.

**Figure 4:**
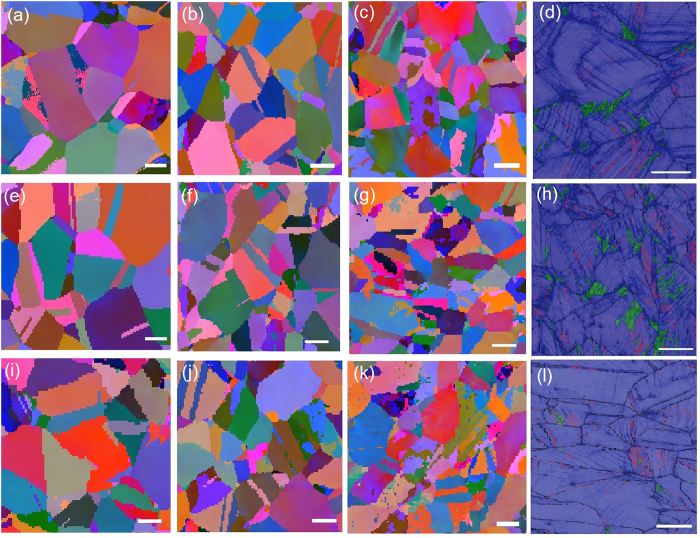
EBSD images showing the fractographies of the fatigued samples. (**a–c**) Images corresponding to the initiation (the mirror region in supplementary Fig. 2b), steady-state propagation (the mist transition region), and final (the hackle region) stages of crack propagation during fatigue fracture. (**d**) A phase map showing the matrix (blue) and the twin (pink), and martensite (green) phases. (**e–h**,**i–l**) Corresponding images for the positively and negatively graded samples, respectively (scale bar: 10 μm).

**Figure 5:**
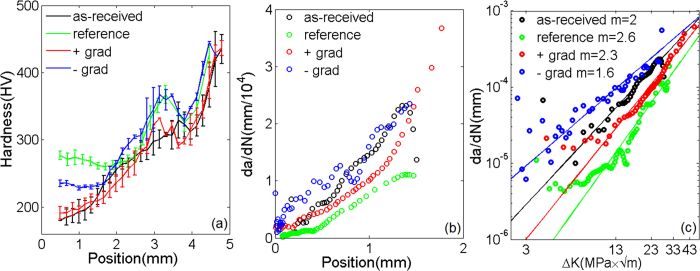
The evolution of the hardness due to fatigue fracture. (**a**) The final hardness as a function of distance from the crack tip. (**b**) The crack growth rate as a function of position. (**c**) The fatigue crack growth rate, d*a*/d*N*, as a function of the stress intensity factor, Δ*K.* The corresponding exponent *m* in the Paris law for four samples is also supplied.

Next, we turned to the EBSD images of the mirror, mist, and hackle regions of a fatigued sample. Figure 4a–c show EBSD images of the fracture surface of the as-received gradient-free sample in the three regions, and Fig. 4d is an amplified view of Fig. 4c that provides phase information. The results revealed a deformation-induced martensitic structure in the final stage of fatigue fracture. The density of the twin boundaries increased as the crack propagated in the as-received gradient-free sample. As the crack propagated, the stress intensity factor, 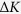, increased, which led to more severe plastic deformation. Because 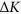 is the primary factor influencing the plastic zone in the as-received gradient-free sample, we expected a gradual increase in the twin density as the crack extended. Figure 4e–g show the microstructure of the positively graded sample in the three regions; phase information for a selected region is shown in Fig. 4h. Similar EBSD images for the negatively graded sample are shown in Fig. 4i–l. In contrast to the as-received gradient-free sample, which exhibited the deformation features shown in Fig. 4a–c, which resulted from isolated fatigue fracture, the microstructures shown in Fig. 4e–g for the positively graded sample and Fig. 4i–k for the negatively graded sample were induced by the combination of pre-torsion and subsequent fatigue fractures. In contrast to their initial states, the graded samples did not exhibit significant differences in twin density until the late stage of fatigue, which was probably due to the small scale plastic deformation that occurred during fatigue fracture. There was a local hardness maximum when the crack growth rate began to accelerate from a steady state, which resulted in more severe plastic deformation, as shown in Fig. 5a. Although a deformation-induced martensitic phase was present in all of the fatigued samples during the late stage (Fig. 4d,h,l), we found that its contribution during the crack initiation and steady-state growth stages was quite limited.

The crack growth rate is shown as a function of the crack tip position in Fig. 5b, from which we deduced the fatigue crack growth rate as a function of the range of the applied stress intensity factor, 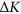. The classical Paris law indicates the following relationship between the crack growth per load cycle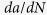 and the stress intensity range:


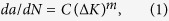


where^22^


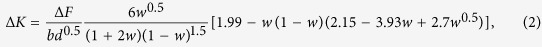


*C* and *m* are material-dependent constants, 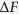 is the amplitude of the force (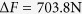), *b* = 2.5 mm and *d* = 5 mm are the sample’s thickness and height, respectively, as shown in Fig. 2b


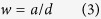


By fitting the above equation to the curves shown in Fig. 5c, we obtained the Paris exponents for the as-received gradient-free, reference gradient-free, positively graded, and negatively graded samples for *m* = *2, 2.6, 2.3,* and *1.6*, respectively. Because the crack growth rate was nearly the same for all four types of sample at the fast fracturing stage, a greater Paris exponent, *m,* actually corresponded to a slower steady-state crack propagation rate (Fig. 5c). This result suggests that, while the reference gradient-free sample had the best crack resistance, positive strength gradients also exhibited superior resistance to fatigue crack extension, in contrast to the as-received and the negatively graded samples (however, negative gradients provide superior resistance to crack initiation).

**Figure 6:**
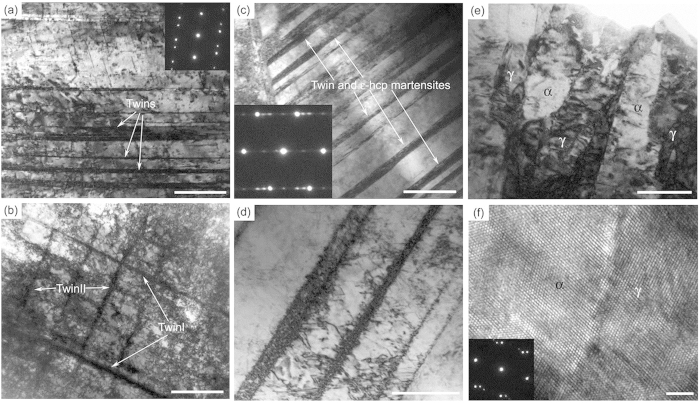
TEM images of the microstructure of the fatigue fractured surface at different stages. (**a**) The mirror region in the negatively graded sample exhibits a high dislocation density. Dislocation activities are confined by twin boundaries. The inset shows the typical electron diffraction patterns of twins (scale bar: 500 nm). (**b**) Second twins with dislocations confined by conjugated twin boundaries in the mist region (scale bar: 1 μm). (**c**) In the mist region of the fatigued positively graded sample, parallel *ε*-HCP martensite and twin boundaries exist. The inset shows an electron diffraction pattern that is typical of both twin and *ε*-HCP martensite (scale bar: 500 nm). (**d**) Parallel HCP and twin boundaries that serve as barriers to dislocations (scale bar: 200 nm). (**e**) During the fast fracture stage in samples of all three types, *α* martensite on the order of tens to hundreds of nanometers in size is common (scale bar: 100 nm). (**f**) A high-resolution image of an austenite-martensite phase boundary. The inset shows an electron diffraction pattern that is typical coexisting of *α* and *γ* (scale bar: 2 nm).

Because information about the twin density along the fracture plane was insufficient to identify the mechanisms behind the distinct fatigue behaviour of the graded samples, we performed observations using transmission electron microscopy (TEM) to determine why the negatively graded samples experienced more fatigue cycles than the positively graded ones during the crack initiation stage and why the positively graded samples excelled in resisting crack propagation. In contrast to the positively graded and as-received gradient-free samples, the negatively graded samples exhibited high dislocation densities in the mirror region (around the location marked ‘c’ in supplementary Fig. 2b), as shown in Fig. 6a. Because the dislocation activities were confined by twin boundaries, the abundant pre-existing twins and associated hardening mechanisms that interact with dislocations appeared to be the primary causes of the enhanced fatigue performance observed^23^,^24^. In the mirror-to-mist transition region (near ‘d’ in supplementary Fig. 2b), we observed rich dislocations confined in domains divided by primary and secondary twin boundaries, as shown in Fig. 6b. In contrast, the mist region (supplementary Fig. 2a) of the positively graded sample was characterized by coexisting hexagonal close-packed (HCP) phases and twin boundaries; the twin/phase domains were generally parallel (Fig. 6c). The coexistence of HCP and twin boundaries is consistent with the sequence of transformation, 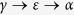, in austenitic steels, where 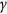 and 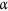 denote the face-centred cubic austenitic and body-centred cubic martensitic phases, respectively, and 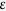 denotes the intermediate HCP phase. The parallel phase and twin boundaries served as barriers to dislocation motion. The confinement of dislocations by the twin and phase boundaries (see Fig. 6d) suggests that the better fatigue resistance to crack propagation observed in the positively graded samples was a result of the reduction in the size of the plastic zone as each fatigue crack propagated. During the late stage, rapid fracture occurred in all of the samples. In addition to dislocation activities and deformation twinning, we observed phase transformations from austenite to martensite. Unlike the results of nanoscale lamellar deformation twinning, these martensitic domains were typically on the order of hundreds of nanometers in size, as shown in Figs 6e and 3d,h,l. Figure 6f is a high-resolution image of an austenite-martensite phase boundary. The presence of a significant number of martensitic structures during the late stage of fatigue fracture suggests that the austenite-martensite transformation may favour higher strain rates in 304 steel. Because the residual life at this point is very short, phase transformation-induced deformation does not seem to contribute much to the overall fatigue performance of graded 304 steel.

**Figure 7:**
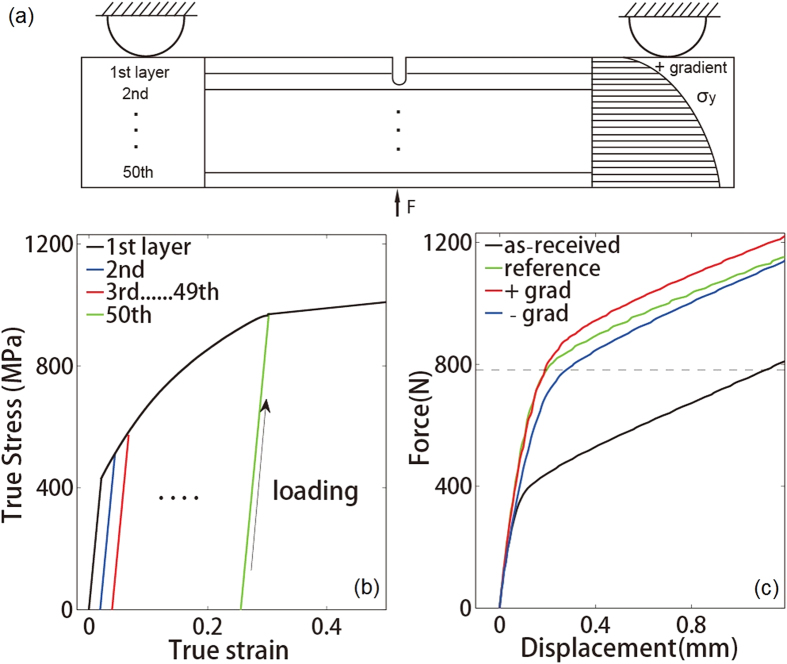
Finite element simulations of the four types of samples during three-point bending tests. (**a**) The model used for the finite element analysis. Materials made up of different layers along the y-axis were assigned different initial strengths. Here, we show a sample with a positive gradient with a round-tipped crack in the side with the lower initial yield strength. (**b**) The material properties of different layers in the finite element model. (**c**) Simulated force-displacement curves for the four types of samples during three-point bending. The dashed line represents the load of 782 N applied in all of the tests.

**Figure 8:**
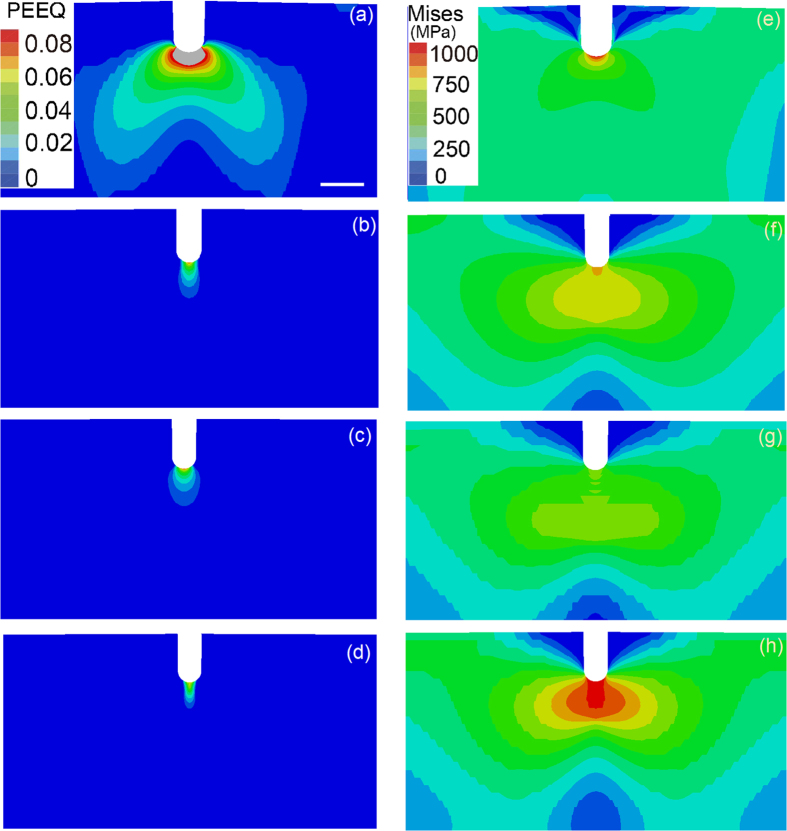
The plastic strain and stress fields at a round-tipped crack-like notch. (**a–d**) Equivalent plastic strain contours at the crack-like notch in the as-received gradient-free sample, the (pre-tension strengthened) reference gradient-free sample, the positively graded sample and the negatively graded sample, respectively. (**e–h**) The von Mises stresses of the previously listed samples (scale bar: 0.5 mm).

**Figure 9:**
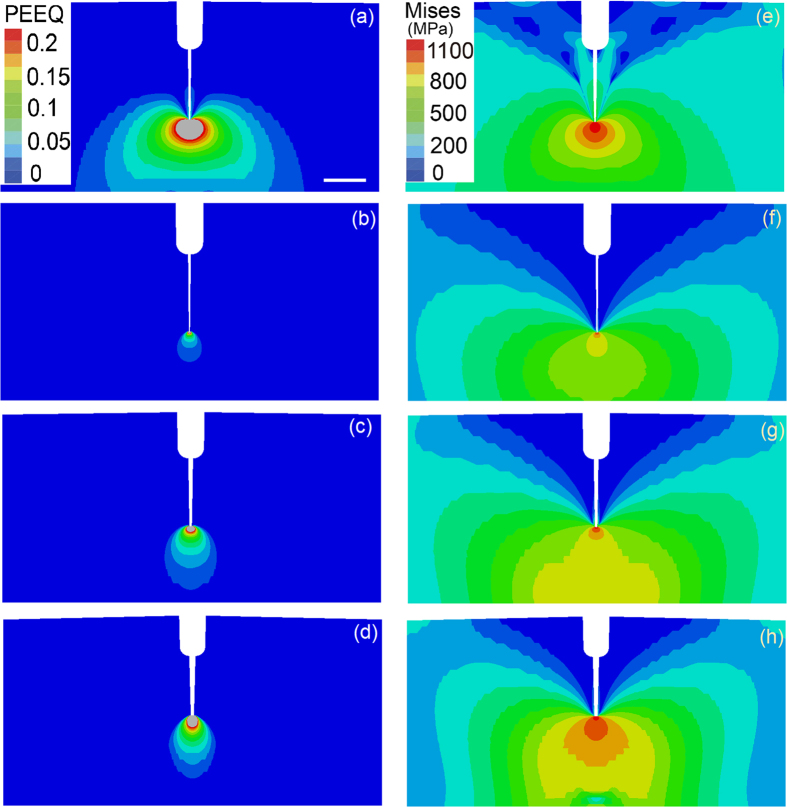
The plastic strain and stress fields near a perfectly sharp crack tip. (**a**–**d**) Equivalent plastic strain contours near sharp crack tips in the as-received gradient free sample, the (pre-tension strengthened) reference gradient-free sample, the positively graded sample and the negatively graded sample, respectively. (**e–h**) The von Mises stress fields of the previously listed samples (scale bar: 0.5 mm).

Furthermore, we conducted a series of finite element simulations to illustrate the influence of the strength gradient on the stress and strain fields of the samples in their initial and partially fatigued states. Initially, the samples contained round-tipped cracks with 0.15 mm root radii, and the tips of the cracks in the partially fatigued samples were assumed to be perfectly sharp. Details of the model are supplied in the Methods section and Fig. 7. For consistency with the load-controlled fatigue tests, we applied the same point force of 782 N to all four types of samples during the three-point bending simulations. The results for the samples that had not undergone fatigue fracture are shown in Fig. 8. We observed that the plastic zone in the sample with a negative strength gradient was the smallest (see Fig. 8a–d). If we zoom in the plastic zone, we see that the plastic strain contour in the positively graded sample, in terms of the maximum value and the size of the plastically deformed zone, is greater than that of the negatively graded sample (supplementary Fig. 3a,b). However, the von Mises stress field in the sample with a positive strength gradient was the most homogeneous, as shown in Fig. 8e–h. These two features correlated with the observation that the negatively graded samples required more fatigue cycles than the positively graded samples for crack initiation. It is noted that the fatigue process could be collectively resulted from the stress, the plastic strain, and local microstructures. A clear fatigue map to differentiate the contribution of individual factors is not available at this stage. Similar trends were observed for the partially fatigued samples, as shown in Fig. 9. The smaller plastic zone size and lower stress concentration in the positively graded sample resulted in slower fatigue crack propagation during cyclic loading in comparison to the other two types of samples.

## Discussion

In summary, the work presented in this paper demonstrates that strength gradients can be exploited to enhance the fatigue behaviour of 304 austenitic steel, one of the most widely used types of stainless steels. We have shown that strength gradients lead to fatigue performance that is better than that of the corresponding gradient-free material, that a negative strength gradient from an existing crack-like notch leads to better resistance to fatigue crack initiation than a positive strength gradient, and that a positive strength gradient is more effective at suppressing crack growth. The impact of strength gradients on fatigue performance is twofold: (a) A strength gradient is a result of a microstructural gradient, and changes in the microstructure could strongly influence local plastic deformation during fatigue crack propagation. For example, Fig. 6a,b show dense dislocation structures stored in the twin boundaries in the negatively graded sample, which could hinder fatigue crack propagation in the vicinity by suppressing further plastic deformation. (b) The strength gradient resulting from a microstructural gradient can significantly alter the stress distribution near a crack tip (Figs 8 and 9), which could also influence the size of the local plastic zone during fatigue crack propagation (Figs 8 and 9). Our finite-element simulations indicated that the samples with negative strength gradients exhibited the smallest plastic zones of all of the samples, while the positively graded samples exhibited the most homogeneous stress fields (Fig. 8), in agreement with the experimental observations. These factors explain why the negatively graded samples have the longest crack initiation lives. Once a crack was initiated, the positively graded samples exhibited smaller plastic zones and stress concentrations than the negatively graded and as-received samples (Fig. 9); therefore, fatigue cracks propagated more slowly in the positively graded samples. Enlarged plastic strain contours for both the positively graded sample and the negatively graded sample are given in supplementary Fig. 4a,b. Now the plastic strain contour in the positively graded sample, in terms of the maximum value and the size of the plastically deformed zone, is smaller than that of the negatively graded sample. Note that graded materials are widely used in engineering applications to provide, e.g., better resistance to thermomechanical loads in aero- and astronautical structures. Various ceramic and metallic alloys have been engineered to include spatial variations in material properties to provide improved performance with low weight and cost^5^,^6^,^7^,^25^. Our study suggests that hardness or strength gradients could substantially enhance the fatigue resistances of materials and calls for further studies to promote both fundamental understanding and practical applications of graded materials and structures.

## Methods

### Mechanical characterization

Commercially available 304 steel primarily consists of 17% Cr, 8% Ni and 72% Fe in weight percentage. As shown in Fig. 1g, the yield strength of as-received 304 steel is approximately 330 MPa. The procedures used to fabricate the graded samples are illustrated in Fig. 2a. First, we twisted the as-received cylindrical samples of 304 austenitic steel through an angle (for dimensions, see supplementary Fig. 1a). The shear strain in the radial direction, 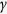, as a result of the pre-torsion, is


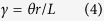


where 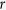 is the radial position and 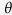 is the net twist in a selected region with a uniform cross-sectional diameter and a length of 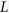 = 60 mm. For a nominal pre-torsion of 180°, the maximum shear strain in a sample with radius 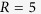
mm is approximately 0.2.

Then, we cut a cylindrical rod from the middle section of the pre-twisted sample with uniform deformation. A rectangular bar was then sectioned from the cylindrical rod. Splitting the rectangular bar from the mid-plane yielded two graded samples. A round-tipped notch with a root radius of 0.15 mm was then cut in the graded samples. Force-controlled loads were applied to the samples during the three-point bending test (Fig. 2c) in the micro static and dynamic testing machine at room temperature and the results were observed using SEM (supplementary Fig. 2c,d). A typical *in-situ* fatigue test required more than 11 hours at a frequency of 10 Hz. The force in each cyclic test varied from 78.2 N to 782 N. From the geometry shown in Fig. 2, we deduced that the amplitude of the nominal stress in the notched plane was


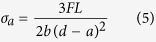


which led to a stress amplitude of 600 MPa^26^. The average yield strength of the graded samples is the same, but its value is higher than that of the as-received samples. Therefore, we also applied pre-tension to several of the as-received bars and controlled the amount of tensile strain so that the strength of the bar after tension would be identical to the average yield strength of the graded samples made from pre-twisted bars. Because the deformation during pre-tension was uniform, the fatigued samples made from the pre-tensioned bars were gradient-free. We labelled these samples ‘reference’ (in contrast to the as-received samples).

### SEM characterization and EBSD analysis

A MIRA3^TM^ (LM) field emission scanning electron microscope from TESCAN was used for microstructure characterization. We used AZtec from Oxford Instruments at 20 kV to perform an EBSD analysis to obtain information on deformation twinning. Scanning the selected region of each image with a step size of 0.5 μm took approximately 20 minutes. At each location, an SEM image was captured in greyscale, and the corresponding EBSD image was captured in colour to reveal different crystallographic orientations. The phase Figures with twin boundaries marked in red were generated from the EBSD images. The austenitic phase is shown in blue, and martensitic phase is shown in green.

### TEM sample preparation

The dual beam focused ion beam/scanning electron microscopy (FIB/SEM) method was used to prepare the TEM samples. After fatigue fracture, the samples were cut in the mirror region (near the location marked ‘c’ in supplementary Fig. 2b), the mist transition region (near ‘d’ in supplementary Fig. 2b), and the hackle region (near ‘e’ in supplementary Fig. 2b).The surface of the specimen was coated with platinum to prevent charging and to reduce damage from the ion beam. A dense beam of Ga+ ions was used to mill deep trenches in the specified regions. Then, foil was prepared in an orientation perpendicular to the crack plane by using the ion beam at an acceleration voltage of 30 kV and a maximum current of 20 pA to excavate on both sides of it. The foil (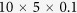 μm) was exhumed from the specimen’s surface using an *in-situ* micro manipulator and transferred to a copper grid. Platinum soldering was used to attach the foil to the copper grid. After being mounted on the copper grid, the foil was ion-milled again to reduce its thickness to 50 nm.

### TEM characterization

TEM observation was performed using a JEM-2100 transmission electron microscope operated at 200 kV. Images were recorded using a charge coupled device camera (2 k × 2 k, Gatan 831) in binning mode two.

### Finite element simulations of crack-tip deformation

We performed finite element simulations to illustrate the influence of the strength gradient on the stress and strain fields of the samples in their initial and partially fatigued states. To represent the strength gradient, we discretized the sample into 50 layers along the crack plane, as shown in Fig. 7. Then, we assigned a yield strength in each layer based on its distance from the crack tip. When the yield strength increased (decreased) with the distance, the sample was positively (negatively) graded. In the positively graded sample, the yield strength of the first layer was 330 MPa, which was equal to the yield strength of the as-received gradient-free sample. In the other 49 layers, the yield strength increased from 356 MPa to 960 MPa (Fig. 7a). As the deformation process continued, although different layers of the material yielded at different stress levels, they all followed the stress-strain curve shown in Fig. 7b. We used the commercial finite-element software package Abaqus 6.11 standard^27^ for all of the simulations. For consistency with the load-controlled fatigue tests, we applied the same point force of 782 N to samples of all four types during the three-point bending simulations^28^,^29^,^30^.

## Additional Information

**How to cite this article**: Ma, Z. *et al.* Strength gradient enhances fatigue resistance of steels. *Sci. Rep.* **6**, 22156; doi: 10.1038/srep22156 (2016).

## Supplementary Material

Supplementary Information
