# Supplementary material for: Strength gradient enhances fatigue resistance of steels
Source: Sci Rep. 2016 Feb 24;6:22156. doi: 10.1038/srep22156 (PMC4764920; doi:10.1038/srep22156)
Supplement: Supplementary Information [file srep22156-s1.pdf]

Strength gradient enhances fatigue resistance of steels

Zhiwei Ma[1], Jiabin Liu[2], Gang Wang[3], Hongtao Wang[2], Yujie Wei[1*], Huajian Gao[4*]

[1]LNM, Institute of Mechanics, Chinese Academy of Sciences, Beijing 100190, P.R. China

[2]Faculty of Engineering, Zhejiang University, Hangzhou 310027, China

[3]Laboratory for Microstructures, Shanghai University, Shanghai 200444, P.R. China

[4]School of Engineering, Brown University, Providence, RI 02912, USA

Correspondences should be addressed to yujie_wei@lnm.imech.ac.cn (Y. W.) and huajian_gao@brown.edu (H.G.).

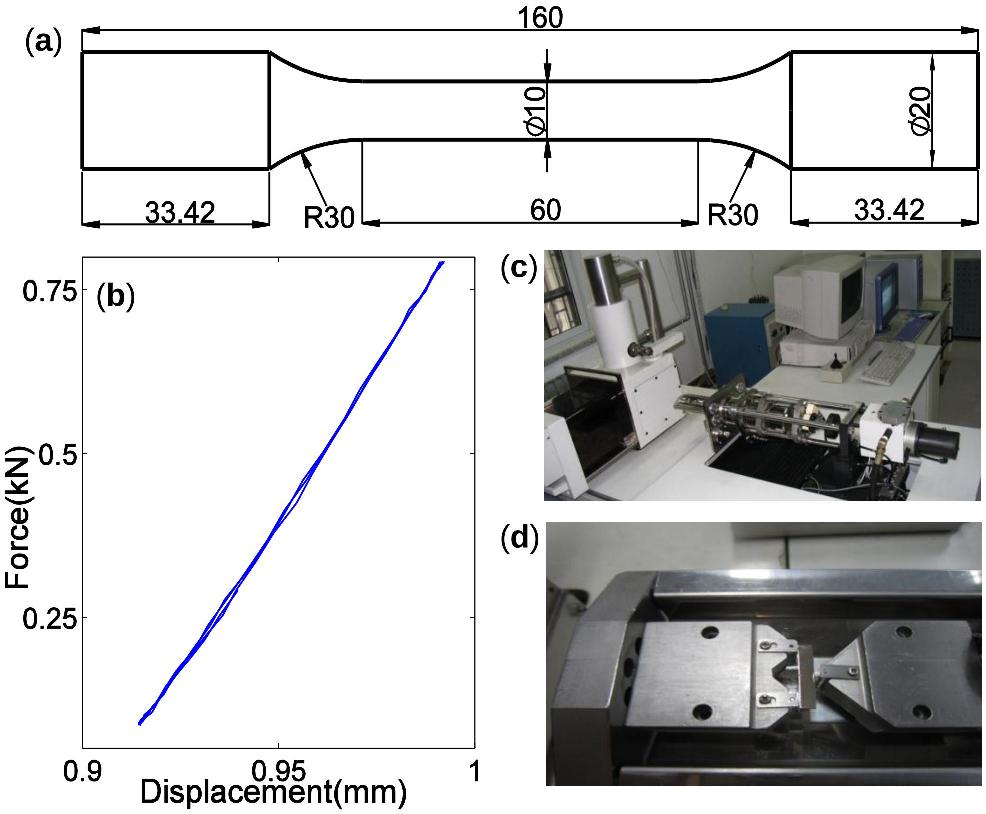

Supplementary Figure 1 | Experimental setup for fatigue tests. (a)Dimensions of the pre-torsion samples (units: mm). For a nominal pre-torsion of 180°, the maximum shear strain in the sample with radius $R = 5$ mm is about 0.2. (b)Applied cyclic force-displacement curves for the three-point bending test. (c) The fatigue testing machine. (d) The amplified part showing the setup of the in-situ three-point bending for fatigue testing.

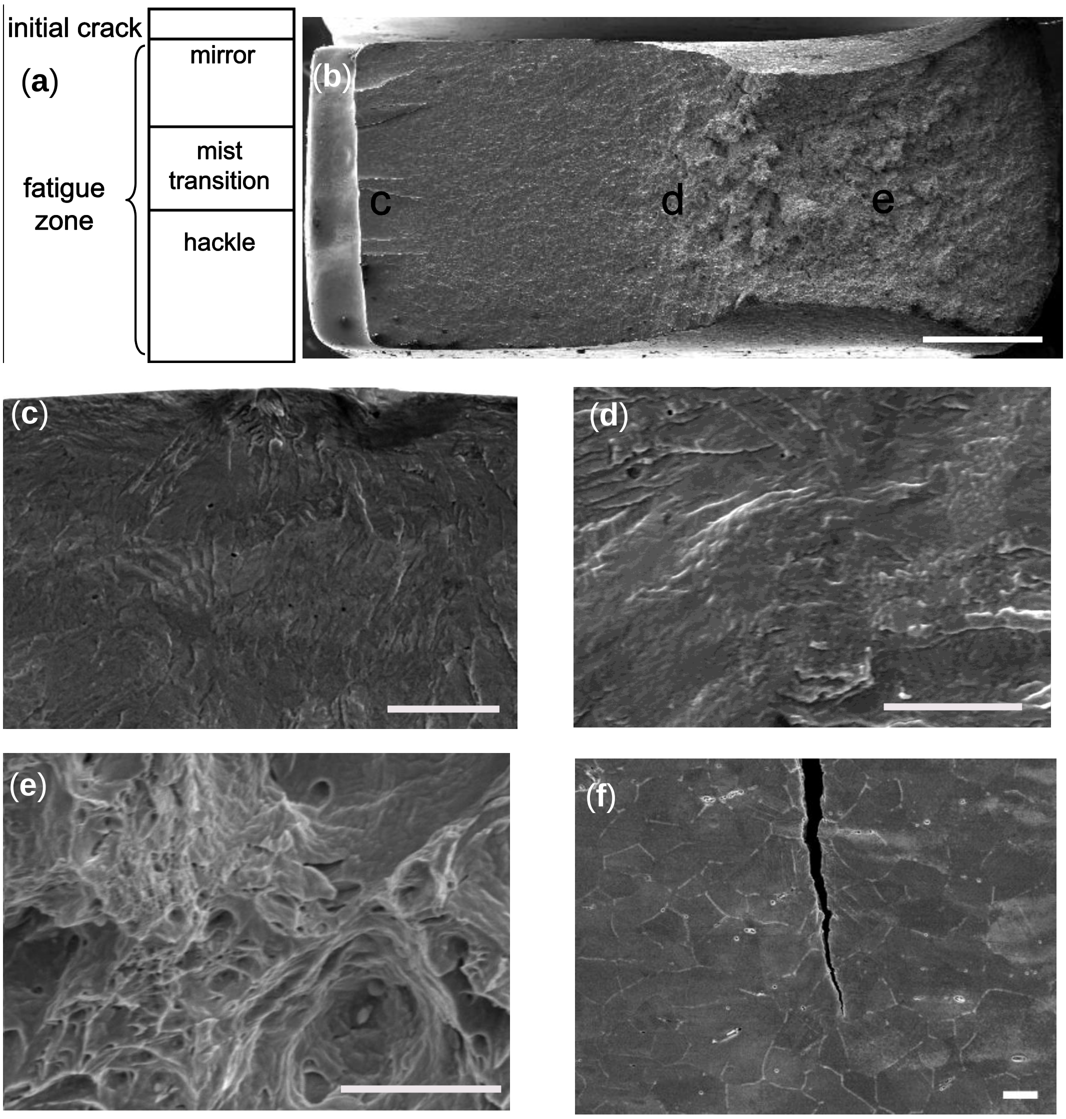

Supplementary Figure 2 | SEM images on fractographies of fatigued samples (600MPa). (a) Schematics to illustrate features of the fracture surface in the four types of fatigue samples. (b) to (f) The fatigue fractured sample with initial negative gradient. (b) Final fractured sample (scale bar: 1.0mm). (c) Microstructures at point 'c' in (b). (d) Microstructures at point 'd' in (b). (e) Microstructures at point 'e' in (b). (f) Side view to show a trans-granular fatigue crack (scale bars in c to f: 10μm).

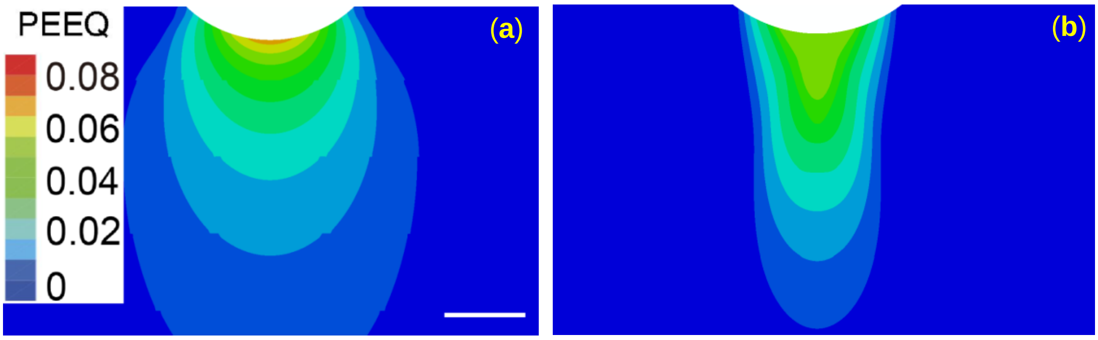

Supplementary Figure 3 | The enlarged equivalent plastic strain contours at a round-tipped crack-like notch. (a) The contour in the positively graded sample and (b) the contour in the negatively graded sample (scale bar: 0.1mm). The plastic strain contour in the positively graded sample, in terms of the maximum value and the size of the plastically deformed zone, is greater than that of the negatively graded sample.

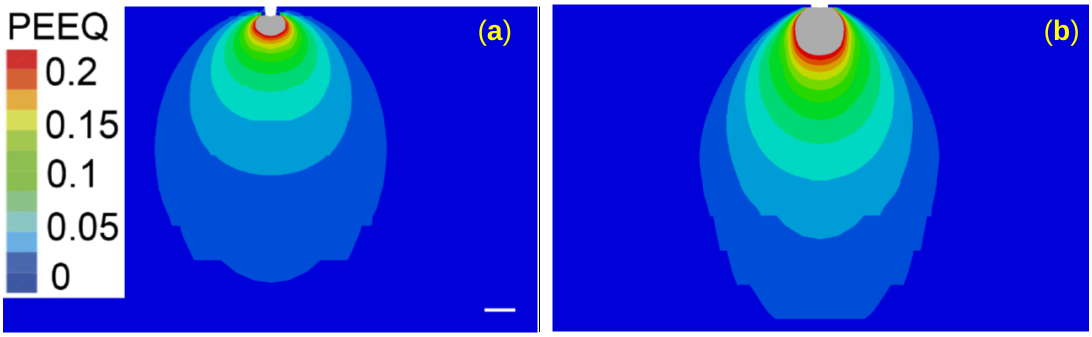

Supplementary Figure 4 | The enlarged equivalent plastic strain contours at the ideally sharp crack tip (after crack initiation). (a) In the positively graded sample and (b) in the negatively graded sample (scale bar: 0.1mm). Now the plastic strain contour in the positively graded sample, in terms of the maximum value and the size of the plastically deformed zone, is smaller than that of the negatively graded sample.
